# Supplementary material for: Volatile Aroma Compound Production Is Affected by Growth Rate in S. cerevisiae
Source: Appl Environ Microbiol. 2022 Nov 15;88(23):e01509-22. doi: 10.1128/aem.01509-22 (PMC9746289; doi:10.1128/aem.01509-22)
Supplement: Supplemental file 1 — Supplemental material. Download aem.01509-22-s0001.pdf, PDF file, 0.2 MB [file aem.01509-22-s0001.pdf]

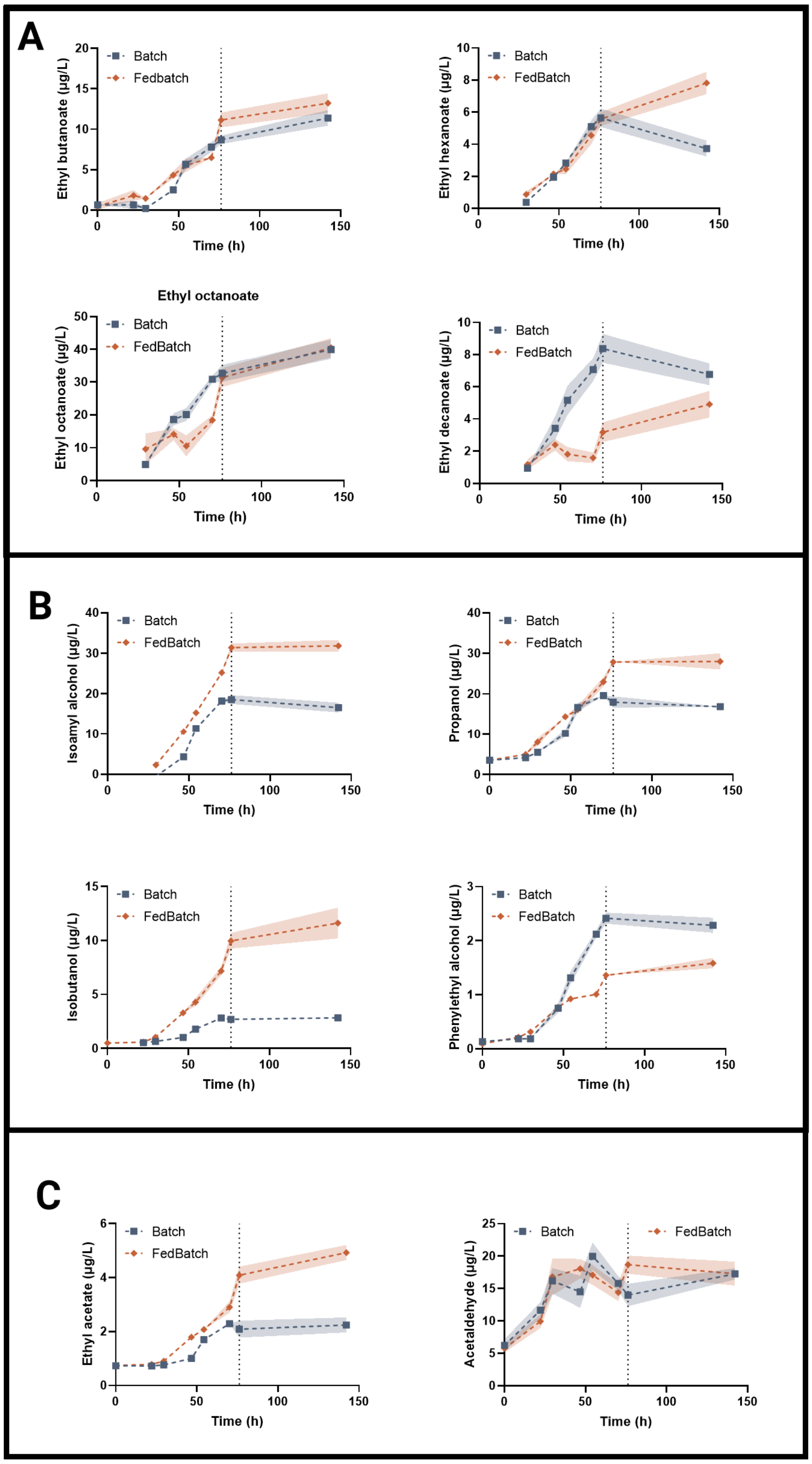

Figure S1 | Production kinetics of (A) fatty acid esters, (B) higher alcohols, (C) ethyl acetate and acetaldehyde in batch and fed-batch fermentations. Standard deviation of two biological replicates is shown in lighter shades in the lines connecting each sample. A vertical dashed line represents the timepoint when glucose was depleted in both batch and fed-batch fermentations, after 76 hours.
